# What does “sustainable seafood” mean to seafood system actors in Japan and Sweden?

**DOI:** 10.1007/s13280-024-02122-4

**Published:** 2025-01-02

**Authors:** Abigayil Blandon, Malin Jonell, Hiroe Ishihara, Aiora Zabala

**Affiliations:** 1 Stockholm Resilience Centre, Stockholm University, Albanovägen 28, 114 19, Stockholm, Sweden; 2 Kungliga Vetenskaps Akademien, Lilla Frescativägen 4A, 114 18, Stockholm, Sweden; 3 Graduate School of Frontier Sciences, 5 – 1 – 5 Kashiwanoha, Kashiwa, Chiba 227-0882 Japan; 4 The Open University, Walton Hall, Kents Hill, Milton Keynes, MK7 6AA UK

**Keywords:** Japan, Q method, Sustainability, Sustainable seafood, Sweden

## Abstract

“Sustainability” can mean different prioritisations of society, environment and economy to different people. As one of the largest globally traded food commodities, for seafood, these differences could have large implications. The study captures different understandings of “sustainable seafood” among 29 key actors along the seafood supply chain—government, NGOs, industry bodies, retailers and producers—using a novel cross-country application of Q method in Japan and Sweden. Sweden, known for its uptake of green consumption, contrasts with Japan’s focus on alternative sustainability initiatives such as *satoumi*. Participants ranked 40 prepared statements on seafood sustainability revealing four distinct perspectives: Regulation-centric, Ecocentric, Industry-centric and Community-centric. There were clear country-based divisions, with only one perspective containing participants from both countries. Interactions and prioritisations of different dimensions of sustainability are also presented, through which we hypothesise areas of conflict and consensus. We stress the need to understand diverse perspectives when tackling global seafood sustainability challenges.

**Supplementary Information:**

The online version contains supplementary material available at 10.1007/s13280-024-02122-4.

## Introduction

Approximately 3 billion people worldwide rely on wild-caught and farmed seafood^1^ as a primary source of protein (FAO 2022). Seafood is also one of the most traded food commodities (FAO 2022) and could have a fundamental role to play in shifting to healthy global diets (Golden et al. 2021). However, seafood production today is faced with multiple dimensions of sustainability problems, including but not limited to overharvesting wild fish stocks (FAO 2022), antibiotic use in fish farms (Naylor et al. 2021a) and modern slavery and human rights abuses (Tickler et al. 2018). If seafood is to meet the doubling in demand forecast for 2050 (Naylor et al. 2021b), it needs to be caught or farmed sustainably.

The concept of sustainability has been interpreted in many different ways (Béné et al. 2019). In their review of sustainability in the context of food, Béné et al. (2019) find that although there is agreement that the current food system is not fit for purpose and something must be done, the reasons for its failure and what should be prioritised for action diverge depending on the communities of practice such as agriculture, nutrition and socio-ecology. In the same way, individuals, organisations and governments may have different perceptions of what constitutes sustainable seafood, and may implement different solutions. For example, Gorjanc et al. (2024) found that different governance actors interpreted the same European marine policy objectives as prioritising either environmental protection or sustainable use, with different actions prioritised depending on actor perspective. Prioritising different dimensions of sustainability also leads to different scenarios for the future of the seafood system (Gephart et al. 2021; Farmery et al. 2022).

This study explores what “sustainable seafood” means for people representing organisations who are associated with production or consumption of seafood. It asks what dimensions of sustainability are prioritised and how these dimensions interact, how seafood is framed and how stakeholders in different country contexts differ or coincide in their conceptualisation. The importance of better understanding different cultural interpretations of seafood sustainability has been highlighted by earlier work, for example, Cullen-knox et al. (2020) who find that as seafood moves down the supply chain from Australia to China, the definition of sustainable seafood changes. It is also crucial to examine this topic as calls grow for redefining sustainability beyond existing definitions (Kourantidou and Kaiser 2019).

This study focuses on two countries with notable similarities and differences in terms of seafood consumption and sustainability: Sweden and Japan. Consumers in both countries have significant spending power while also enjoying representative governments and liberal democracies. Despite these commonalities, the countries appear to be notably different when it comes to how seafood sustainability is understood. One example is the awareness and uptake of ecolabels such as the Marine Stewardship Council (MSC), the market leading certification standard for sustainable wild capture fisheries (Bush and Oosterveer 2019). Sweden and Japan appear to be at opposite ends of the global spectrum, with Swedish consumers showing one of the highest awarenesses of MSC, and Japanese consumers the lowest (GlobeScan 2020). An IPSOS survey undertaken in 2019 also shows that Japan has a distinct lack of interest in typical ecological sustainability issues surrounding seafood compared to other countries (IPSOS 2019). We, therefore, assume investigating these two countries will uncover a range of conceptualisations of sustainable seafood applicable for other countries lying somewhere in-between these extremes.

### Case study background

#### Japan

Japan is one of the world’s four major markets for aquatic foods (FAO 2022). Although it is one of the top five seafood consuming countries in the world (FAO 2022), domestic consumption of seafood is on a decreasing trend, and has dipped below meat since 2011. In 2021, per capita annual consumption was 23.2 kg (JFA 2022).

In terms of sustainability, although Japan was the first country in Asia to certify a fishery with MSC in 2008 (Blandon and Ishihara 2021), the growth is slow, with currently 12 fisheries certified with MSC and 82 farms certified by the Aquaculture Stewardship Council (ASC), the equivalent certification scheme for farmed fish (ASC Japan 2022). There are also, however, examples of domestic initiatives such as *satoumi*, or more recently *umigyo*, that highlight the alternative ways of approaching sustainability (Mizuta and Vlachopoulou 2017), focussed more on production rather than consumption. *Satoumi* can be defined as a “coastal area where human intervention increases biological productivity and diversity” (Yanagi 2012) and was developed from the concept of *satoyama*—the equivalent concept for an agricultural area. *Umigyo* is a concept adopted by the Japanese Fisheries Agency in their Fisheries Basic Plan in 2022 which represents activities that increase income opportunities for fishing communities by using the value and beauty of the sea and fishing villages, one pillar of this being sustainable seafood production (JFA 2023).

Japanese coastal fisheries management has historically centred around co-management—a combination of top-down and bottom-up approaches at the bay level, where fisheries associations would manage the fisheries while territorial use rights are granted by the national government (Makino 2011; Ganseforth 2023). A recently enacted Fisheries Reform will, however, lead to an increase in the total number of species subject to the Total Allowable Catch quotas (TAC), calculated the achieve the optimal level of fishing, and the introduction of Individual Quotas, the distribution of the TAC among individuals based on predetermined rules (Hirokawa and Thompson 2023). It is a typical method of top-down output control, where fishers are controlled according to how much they catch, rather than how much activity they are allowed (input control). This has been met with criticism from proponents of traditional management approaches (Ganseforth 2023), which include communally decided input control measures such as no-take zones, fishing seasons and gear types.

#### Sweden

In contrast with Japan, Sweden is a small producer and consumer of seafood. In 2019, seafood consumption in Sweden was estimated at 12 kg per person annually (Hornborg et al. 2021), about half of Japan’s consumption and below the Swedish Food Agency’s dietary recommendation of eating seafood 2–3 times per week (Hornborg et al. 2021).

Currently 25% of all seafood sold in Sweden is eco-certified (Hornborg et al. 2021), the main certifications used being the MSC and KRAV, focusing on organic production (Nøstvold et al. 2013). Consumers have a 62% rate of recognition of the MSC ecolabel (GlobeScan 2020) indicating prevalence within supermarkets. As well as certifications, most Swedish retailers and value chain actors rely on the World Wildlife Fund (WWF) traffic light list^2^ to judge which species are sustainable (Nøstvold et al. 2013). The Swedish seafood system, therefore, relies heavily upon market-based tools to determine sustainable sourcing (Hornborg and Axelsson 2023).

Since Sweden joined the EU in 1995, fisheries are regulated by the Common Fisheries Policy (CFP). The CFP uses the TAC system of management for managing commercially important species. The TAC is set through a negotiation process between EU member states, which means that often the quota is set much higher than the scientific advice (Aps et al. 2007). The quota for Sweden is distributed into individually transferrable quotas (ITQs) for each fishery vessel. This has led to a consolidation of vessels into fewer, larger vessels that can make more profitable use of the ITQ (Waldo and Paulrud 2013).

In both Japan and Sweden, aquaculture is a small industry compared to wild capture fisheries. In Japan, it accounted for only 0.6% of production volume in 2020 (JFA 2021), while is Sweden it accounted for 6.8% of live weight seafood production in 2022 (Ericson 2023; Leonardsson 2023).

The two countries show distinct differences in the emphasis placed on eco-certifications as an approach for sustainability: Sweden relies heavily on certifications whereas Japan places value on alternative approaches. As well as this, there are large differences in governance arrangements, with Japan having a strong history of co-management whereas Sweden’s management is top-down, from the EU level. By examining these two cases, the study provides valuable insights into how seafood sustainability is perceived and implemented under different cultural and regulatory conditions which may also exist in other countries.

## Theoretical framework

One of the most common ways sustainability has been conceptualised is the systems approach, where sustainability is split into social, economic and environmental dimensions (Giddings et al. 2002). The systems approach to sustainability has been applied to fisheries in a variety of ways (e.g. Stephenson et al. 2018), although there are criticisms that the ecological pillar is over-emphasised (Garlock et al. 2022). Stephenson et al. (2018) suggest that there is a relative lack of attention being paid to social aspects of sustainability such as communities and well-being. Trade-offs between the three dimensions are a key point of debate: Meta-analyses have shown that economic, social and ecological management objectives are likely to be mutually reinforcing (Asche et al. 2018) while another analysis found that existing models of fisheries management, that prioritise conservation, are insufficient to achieve sustainability across all dimensions (Garlock et al. 2022). The perspectives and priorities of key actors in the seafood system in relation to these dimensions of sustainability becomes important in the implementation of effective sustainability approaches.

Framing sustainability issues is crucial, as it influences how events and situations are interpreted by emphasising certain aspects of the problem (Entman 2004). From a sustainability perspective, the three dimensions are a common conceptualisation, but from a food systems perspective, there are alternative ways to frame seafood, for example, as a “commodity”, as a “human right” and as a “common good”. Food is framed as a commodity when it is treated as a tradable good based on its economic value, and is typically prevalent within economic growth narratives, treating people as consumers rather than citizens. This has similarities to the economic dimension of sustainability. When food is framed as a human right, it implies fair, transparent access to food production, the absence of human and resource exploitation and can manifest as mandatory criteria for public procurement or food waste. Although both framings have similarities to the social dimension of sustainability, the human right framing’s main difference with the common good framing is that the state can act as the main guarantor for the right to food and production. Food framed as a common good implies instead a more decentralised or polycentric governance of food embedded in regional contexts, and is focussed on the communal management of food resources. The three framings are not mutually exclusive but do differ in terms of the emphasis placed on different aspects of the food system and were investigated by Jackson et al. (2021) as narratives within the move towards a sustainable food system.

## Materials and methods

### Q method

In this study, we use Q method to investigate participants’ conceptualisations of sustainable seafood. Q method is a tool to analyse conceptualisations of complex phenomena. Participants are asked to rank statements that relate to the phenomena according to how much the statements align with their views. Q method does not require a certain number of participants (Watts and Stenner 2014), the participants are chosen to draw out the *different* viewpoints that exist, rather than the *representative* viewpoints. The Q sorts, the ranking of the statements by each participant, are analysed using multivariate techniques to extract groups of similar Q sorts which represent different ways of thinking about the topic. The steps to design a Q study include (1) creating a Q-set—a set of statements representative of all possible viewpoints on the topic, (2) creating a sorting exercise where participants will sort the statements into a predefined grid ranging from “most agree” to “most disagree”, (3) inviting participants to undertake this Q sort exercise and (4) analysing the ranking of the statements using statistical tools and interpreting this together with the interview data.

### Q-set and Q method design

Our question to participants was “What are the important issues in achieving sustainable seafood?” To create a Q-set of statements, we mapped organisations associated with the seafood sector within both countries (see Table S1), searched their websites and gathered relevant material: reports, blogposts, webpages, position statements, responses to government consultations, etc.

The first author reviewed all material, and inductively collected and adapted statements based on the viewpoints expressed, until saturation. In total, material was collected from 20 Swedish and 16 Japanese organisations, resulting in 113 statements from Swedish sources and 176 from Japanese sources (the Q concourse).

The first author translated the Q concourse into English, reduced the number of statements to 72 and reworded them iteratively, following Q method statement development standards (Watts and Stenner 2014), such as clear, concise phrasing and avoiding multiple propositions or qualifications. The co-authors then validated the statements and reworded or removed them based on their expertise. The statements were categorised into those that address ecological, social and economic sustainability, and also how the statement framed seafood: as a commodity, common good, human right (three categories from Jackson et al. 2021) or as “biosphere-centric”, a category that appeared inductively from the data. The rationale behind the categorisation is given in the Supplementary information (Table S3). The proportion of statements in each category was kept largely balanced to generate a balanced Q-set. We undertook 9 pilot Q sorts (excluded from the final analysis) with researchers and stakeholders who work closely with the seafood industry in both countries, revising the statements based on their feedback.

The final number of statements was 40, which we judged was a manageable number of statements for participants to sort during an hour interview, covering key topics in both countries. We then translated the statements back into Japanese and Swedish, with a back-and-forth iteration to ensure consistency and clarity. The final Q-set, including categorisations, is available in Table 2, with the Swedish and Japanese translations in the Supplementary information (Table S2).

**Fig. 1:**
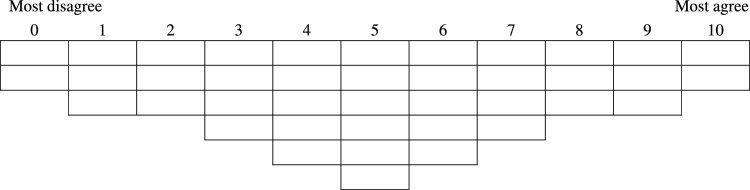
Q sort grid used in this study

The Q method was administered both in-person and online via Zoom. The code used to set up the survey online can be found here.^3^ We used a broad Q sort grid (Fig. 1), with choices from 0 to 10 (most disagree to most agree).^4^

### Q sorting and structured interviews

Each participant was invited to an hour-long virtual or in-person meeting with the first author and in most cases, a co-author. Participants were asked to undertake the Q sort with the following conditions: (1) to answer based on their own opinion, not their organisation’s, and (2) to consider seafood that was produced or consumed in the country where they were based (i.e. either Sweden or Japan). After the Q sort, a structured interview followed, where the participant and interviewer(s) discussed the Q sort. The interview guide is available in the Supplementary information (p12).

We approached individuals from various roles related to the seafood industry, including those from aquaculture companies, feed companies, fisheries companies, industry bodies (representatives of fisheries and aquaculture production), seafood distribution companies, food companies (those using seafood as ingredients to produce food products), retailers, government agencies and NGOs. We began with known contacts and used snow-ball sampling to identify additional interviewees who worked in different positions along the supply chain. While our participants included several primary producers (director of the fisheries company and those working in aquaculture, Table 1), we intentionally prioritised voices from seafood industry organisations that represent a broader spectrum of producers rather than focusing solely on individual producers. Although we did not ask participants for opinions representative of their workplace, we believe that selecting individuals from various positions along the supply chain allowed us to gather diverse perspectives on the seafood system. We collected Q sort data from 29 participants in total, 14 from Japan and 15 from Sweden (see Table 1).

**Table 1:** Workplaces of Q sort participants and their roles within their organisation

Stakeholder type	Japan (number of participants)	Role(s) in organisation	Sweden (number of participants)	Role(s) in organisation
Aquaculture company	2	Production Management Section Assistant Manager (fisheries cooperative association), Quality Control Section (aquaculture company)	1	Head of aquaculture company
Distribution	1	Managing Director (wholesalers association)	2	Training coordinator (wholesalers), Director (fish distribution company)
Feed company	1	Sales and Consulting (global feed company)	–	
Fisheries company	1	Director (fisheries company)	1	Head of Sustainability (fisheries company)
Food company	3	Director of Sustainability Promotion Department (food company), Corporate Social Responsibility Section Manager (food company), Sustainability Group General Manager (food company)	1	Research and Strategic Partnership Manager (food company)
Government	1	Marine Biodiversity Survey Specialist	3	Head of Unit, Aquaculture Coordinator, Fishery Coordinator
Industry body	2	Deputy Director of Fisheries Policy Department (fisheries cooperative association), Researcher at the Fisheries Policy Department (fisheries cooperative association)	3	Expert (fisheries federation), Business Manager (seafood marketing organisation), Head of seafood trade organisation
Environmental NGO	2	Fisheries Resources Management and IUU Policy Manager (NGO), Head of NGO	2	Expert in Fisheries and Market (NGO), Expert in Marine Ecosystem and Fisheries (NGO)
Retailer	1	Sustainability Manager (food retailer)	2	Category Manager (food retailer), Sustainability Strategist (food retailer)
Total	14		15	

### Analysis

The statistical programme R and package “qmethod” (Zabala 2014) was used to analyse the results. The package calculates the correlation matrix between each Q sort, then uses principal component analysis and varimax rotation to group the Q sorts into factors containing similar Q sorts. The number of factors, or perspectives, that were extracted from the data was decided considering a number of criteria suggested in the literature (Watts and Stenner 2014): (1) satisfying Humphrey’s law for all factors, (2) having an associated eigenvalue above 1 for all factors and (3) having the total percentage of variance explained by all factors to be at or above 35–40%. We also considered the rule of thumb of extracting one factor for every 6–8 participants and the scree plot of eigenvalues for each factor to see when the line changes slope (Watts and Stenner 2014).

Once we extracted the factors, we manually removed two Q sorts loaded on factors that should not have been and repeated the analysis. Idealised Q sort patterns representing each perspective were estimated from the analysis. The first author used the Watts and Stenner (2014) crib sheet technique to interpret the factors. Statements that were treated significantly differently (distinguishing statements) and significantly similarly (consensus statements) were determined from analysis. The interpretation of the factors is presented as narratives in Sect. “Four emerging perspectives”, incorporating the important Q statements of each factor and their positioning in the idealised Q sorts.

If the participants consented to the interview being recorded, these were transcribed using Whisper (OpenAI 2023) and any quotations relating to the statements in the Q-set were extracted. We referred back to these quotations during our interpretation of the factors, to add more understanding and nuance.

## Results and discussion

Within this section, we first discuss the quantitative characteristics of the factors extracted in Sect. “Factor extraction”. We then present narratives for each factor in Sect. “Four emerging perspectives”. Following this, we expand on a number of discussion points that we found significant during our analysis: the differences of opinion between and within countries (Sect. ”Differences between countries” and “Differences within countries”); participant perspectives on ecological aspects and regulations (Sect. “Ecological aspects and restrictive regulations”) and areas of consensus (Sect. “Areas of consensus”). Finally, we touch on limitations of the study (Sect. “Missing viewpoints and other caveats”) and our contributions to the literature (Sect. “Cross-country and cross-dimensional comparisons”).

### Factor extraction

Based on the criteria explained in the methods, we extracted four factors explaining 59% of the total data variance. The criteria for the number of factors extracted given in Sect. “Analysis” were met (see Supplementary information Table S4), and the amount of correlation between each factor shows that they were distinct perspectives (Table S5). In order to check the variation in opinion within participants from the same country, we conducted a sensitivity analysis which can be found in the Supplementary information.

The idealised Q sort patterns for each of the four factors are provided in Table 2. The factors are generally split clearly along country lines, with Factor 1 being represented only by Japanese participants, and Factors 2 and 3 only by Swedish participants. Factor 4 is represented by respondents from both countries. There were five participants whose Q sorts were not loaded onto any factor. Four statements distinguished all factors from each other (8, 14, 18 and 26), and two statements were of consensus across all factors (7 and 11). We developed an identity for each factor: Factor 1 represented a regulation-centric perspective, Factor 2 represented an ecocentric perspective, Factor 3 represented an industry-centric perspective and Factor 4 represented a community-centric perspective (see Sect. “Four emerging perspectives”).

**Table 2:**
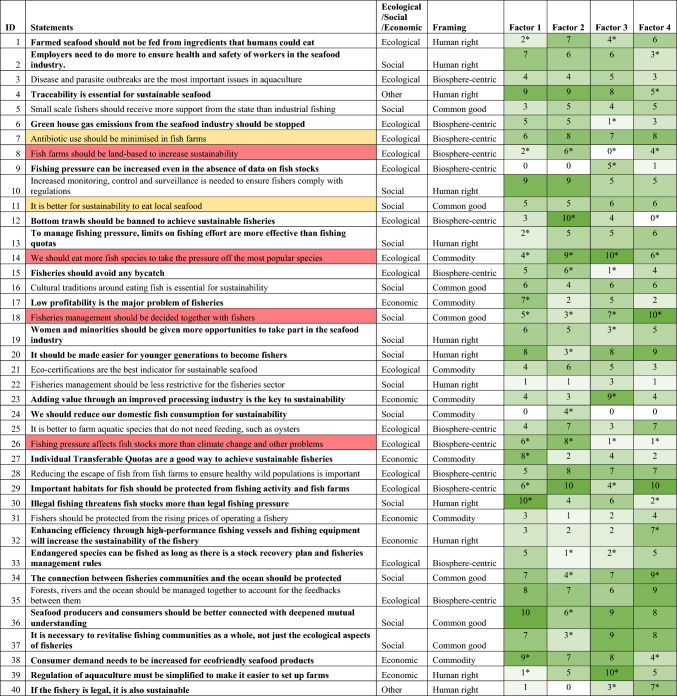
Q-set of 40 Q sort statements used in the study, categorised into types of sustainability (ecological, social or economic) and seafood framing (based on Jackson et al (2021)). Idealised Q sort pattern for each factor (from a range of 0–10) shown in the last four columns. The greener the colour, the higher the ranking of the statement and the higher the strength of agreement the factor has with the statement. Statements with the greatest disagreement between factors (distinguishing statements) are marked in red, and those with the greatest agreement between the factors (consensus statements) are marked in yellow. Statements that some factors feel strongly about, while others are neutral, are marked in bold. Idealised Q sort pattern figures with an asterisk indicate that the statement was a distinguishing statement for that factor

### Four emerging perspectives

An overview of each factor is given in Table 3, and summary narratives are given below, using the crib sheet technique and the qualitative interview data to help with interpretation. The narratives are linked to the Q statements in brackets, with the first number being the statement ID number given in Table 2 and the second number being the column where the statement is placed in the idealised Q sort for that factor. The quotations are pulled directly from interview data and are from participants that fall under each perspective.

**Table 3:**
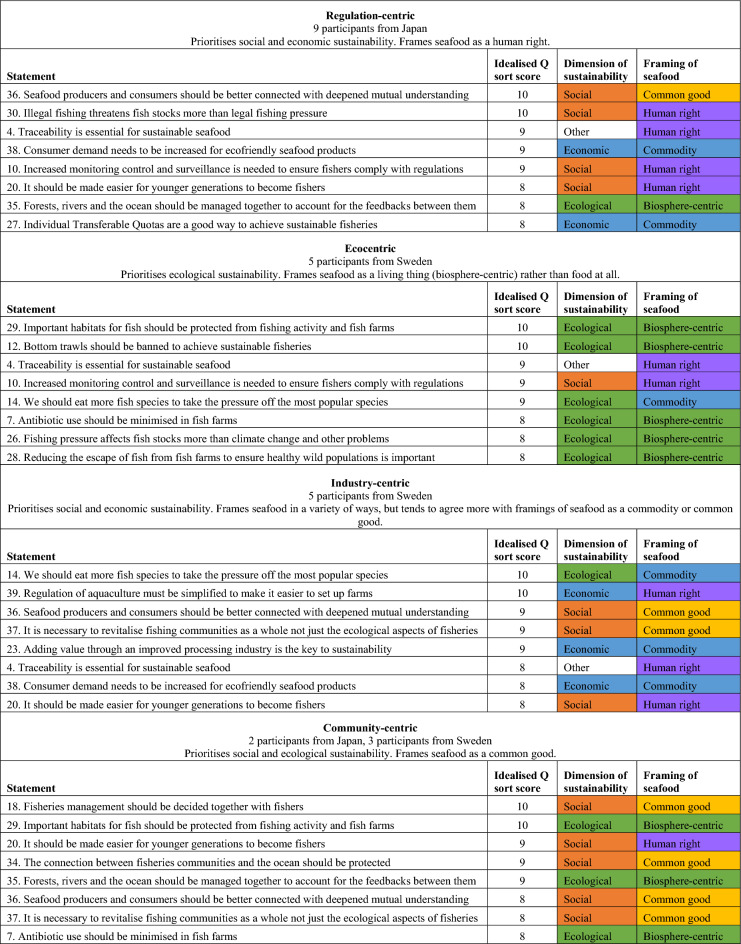
Summary of the four factors including their name, number of participants represented and top 8 statements according to the idealised Q sort pattern, with the categorisations of the statements into dimensions of sustainability (ecological, social and economic) and framing of seafood (based on Jackson et al. (2021)). The categorisations are colour-coded in order to provide an easy overview of the patterns in each factor: dimensions of sustainability are coloured green (for ecological), orange (for social) and blue (for economic), framings are coloured blue (for commodity), purple (for human right), yellow (for common good) and green (for biosphere-centric). Economic sustainability and a commodity-framing, and ecological sustainability and a biosphere-centric framing, were considered similar perspectives and, therefore, given the same colours

#### Factor 1—Regulation-centric

Nine participants were significantly associated with this factor, all from Japan.

This factor prioritised social and economic sustainability issues over ecological issues, and primarily framed seafood as a human right.

In general, this factor emphasises the need for technical, state-led policy fixes to the current fisheries management, alongside some more social, community-centric aspects.

For example, the highest scores were given to the impact of illegal fishing (#30: + 10) and the need for connection between producers and consumers to improve mutual understanding (#36: + 10). In order to solve these issues, the importance of traceability (#4: + 9), monitoring control and surveillance (#10: + 9) and consumer demand for eco-friendly seafood (#38: + 9) were emphasised.

Some of the regulation and policy-related statements significantly distinguished this factor from others. For example, Individual Transferable Quotas (ITQs) were seen as a good way to manage fisheries sustainably (#27: + 8), and input controls were not perceived as better than quotas as a way of managing fishing pressure (#13: + 2). In general, more control and predictability were considered important, as articulated by Participant 1 on statement 10: *“If we were able to manage data on fish landings or bycatch in real time … then we would be able to make the [fisheries] resource assessment cycles shorter which would allow for more appropriate management”.*

The governmental policy orientation of the factor is also emphasised by the statements that were disagreed with, with many statements around reducing restrictions being placed in the lowest columns. For example, fishing when there was no data on stock status was given the lowest Z-Score (#9, 0), while less restrictive fisheries management (#22, + 1) and simplified aquaculture regulations (#39: + 1) were also disagreed with. Participant 18 noted (on statement 9)*“If you don’t have any data on [the fish stock], then you don’t know how many fish there are, so this becomes a problem when considering ideas like sustainability”.*

#### Factor 2—Ecocentric

Five participants were significantly associated with this factor, all from Sweden.

This factor prioritised ecological sustainability over economic and social sustainability, and accordingly framed seafood in a biosphere-centric way (rather than as food at all).

The highest scores were given to ecological sustainability issues, such as banning bottom trawls (#12: + 10) and protecting important habitats for fish (#29: + 10). Fishing pressure was seen as the major factor affecting fish stocks rather than climate change and other factors (#26: + 8). In aquaculture, antibiotic use (#7: + 8) and fish escapes (#28: + 8) were seen as major issues that should be minimised. Fishing endangered species, even under management rules, was strongly disagreed with (#33: + 1). In general, this factor prioritised the health of the ecosystem and the fish over the industry or social interests.

In terms of policy and interventions, the factor agreed with more stringent regulations and higher levels of enforcement. Traceability (#4: + 9) and monitoring, control and surveillance (#10: + 9) were emphasised as important, as well as increasing restrictions on fisheries (#22: + 1), and increasing fishing pressure in the absence of data was placed in most disagree (#9: 0). The current definition of a legal fishery, such as the regulations around fisheries quotas, was not seen as enough to ensure sustainability (#40: 0):

*So we are increasing this quota even though there is really no room to increase it.... herring fishing …, cod fishing has for many years remained within the framework of these multi-year plans that have existed in the Baltic Sea. And despite this, the stock has declined and crashed. So it does not always help that it is legal. Because the stocks have been decided by people. And people have political luggage. (Participant 27 on statement 40)*

ITQs were rejected as an approach that works well to manage fisheries sustainably (#27: + 2). On the other hand, one alternative to ITQs—a more participatory approach involving fishers in the decision-making—was also ranked low (#18: + 3) as there was some wariness of allowing fishers to manage their own fishing pressure. In general, social aspects of seafood and fisheries were not seen as important or relevant within the work to reach seafood sustainability (#20: + 3; #37: + 3; #16: + 4; #34: + 4). Statements framing seafood as a commodity or a common good are also ranked lower in this factor.

#### Factor 3—Industry-centric

Five participants were significantly associated with this factor, all from Sweden.

Similar to factor 1, this factor prioritised economic and social sustainability over ecological sustainability. The factor used a mixture of framings but tended to agree more with framings of seafood as a commodity or common good.

The most important aspects of sustainable seafood were the social and economic longevity of the seafood industry. Issues such as importance of revitalising fisheries communities as a whole (#37: + 9), eating local seafood (#11: + 6) and cultural traditions around eating seafood (#16: + 6) were emphasised in comparison with the other factors. Strategies to move towards sustainability centred around economic and industry sustainability, rather than industry restrictions. A key strategy for sustainability was increasing the diversity of seafood that we eat in order to reduce pressure from the popular species (#14: + 10). Another important strategy was the improvement of the processing industry so that value could be added to currently under-utilised fish species or parts of fish, in order to use what is caught sustainably (#23: + 9).

*I think we cannot just catch [more, but] we should maximise the use of [what we catch] just as we did in the past. Or like when you kill a cow, that you use every little thing. That you do not just throw it away or burn it. (Participant 28 on statement 2)*

There was opposition to restrictions on the seafood industry. Aquaculture regulations were perceived to be too restrictive to set up farms (#39: + 10) and in comparison with other factors, there was more tendency to believe that fisheries management should be less restrictive (#22: + 3).

*[T]oday we have a lot of regulations. We have very many authorities [with] many different functions [...] that are responsible for different types of regulations... [T]he system we have built up in Sweden today is incredibly inefficient. And then, we have a lot of over-implementation of EU regulations as well. (Participant 22 on statement 39)*

In comparison with other factors, statements to do with ecological sustainability which frame seafood in a biosphere-centric way were ranked low (#6: + 1; #15: + 1; #29: + 4).

#### Factor 4—Community-centric

Five participants were significantly associated with this factor. Two of the participants were from Japan, and three of the participants were from Sweden.

This factor prioritised social and ecological sustainability over economic sustainability, and tended to frame seafood as a common good.

This factor placed importance on the wider environment and community within the seafood industry. There were an abundance of social sustainability factors that emphasise the local community and seafood as a common good in the highly ranked statements. The inclusion of the fishers in decision-making was seen as of particular importance (#18: + 10), linking to the communal management idea within the common good framing, as well as the connection between fisheries communities and the ocean (#34: + 9) and the encouragement of the younger generation to become fishers (#20, + 9). From an ecological point of view more holistic solutions such as protection of important habitats for fish (#29: + 10), and management of forests, rivers and the ocean (#35: + 9) were highly ranked.

Economic factors and the framing of seafood as a commodity were given low importance. Consumer demand for eco-friendly seafood products was not prioritised (#38, + 4) and eco-certifications were not seen as the best method to reach sustainable seafood (#21: + 3), representing a lack of buy-in to current market-based approaches to sustainability.

### Differences between countries

One of the main results of this study is the clear distinction between perspectives representing the Japanese participants and Swedish participants. The three groups which explained the highest percentage of data variance were single country groups. This was predictable given the different country contexts that exist in Sweden and Japan when it comes to their seafood industry, as described in the introduction.

The geographical distinction between perspectives can be explained through current debates on seafood sustainability. For example, current discussions in Japan about the implementation of the Fisheries Policy reform mean that output control, such as ITQs, vs input control are on the top of the agenda. People are also aware of the need for traceability due to the new policy to reduce illegally caught seafood in supply chains in Japan (Hirokawa and Thompson 2023), and therefore prioritised this in their answers. In contrast, participants from Sweden answered more neutrally to questions about input vs output control (statement 13) or the threat of illegal fishing (statement 30). This may be due to lack of media attention, as well as the fact that fisheries management is decided at an EU level that participants may perceive is harder to change. Participant 24 explained: “*We don’t have much autonomy over our fish stocks; it is done at the EU level*”. In contrast, the issue of bottom trawling, which has been discussed widely in Swedish media,^5^ splits the Swedish participants.

In terms of aquaculture, in Sweden, the industry is struggling to grow, in part due to the complicated and lengthy legal process (Kyrönviita 2022). This was supported by the high ranking of statement 39 “Regulation of aquaculture must be simplified to make it easier to set up farms” by both Ecocentric and Industry-centric groups, whereas it was ranked lower in the Regulation-centric group, since aquaculture in Japan is tending the other way towards deregulation (Ganseforth 2023).

These differences highlight the context-specific nature of sustainability concepts and how local discussions and media shape perceptions. Variations between countries were particularly evident in how regulatory approaches were perceived. We could imagine that countries with similar regulatory backgrounds to Japan or Sweden may have similar viewpoints on sustainability. Country backgrounds may determine future pathways to sustainability, such as those discussed in Gephart et al. (2021) who split their future aquaculture scenarios based on extent of globalisation and economic growth paradigms.

### Differences within countries

As well as a distinction between countries, the results also highlight differences within countries. As can be seen from the results, the Swedish participants were split along some ideological lines of supporting the environment versus the industry. The differences in the categorisations of groups that were prioritised (ecological, social or economic) are stark between the Ecocentric and Industry-centric groups. It could be interpreted that these two perspectives have arisen due to each other’s existence, with arguments on either side evolving in response to one another. For example, participant 6 commented: *“The worst thing we can get is authorities that just don’t listen [to the fishers] at all. Or are not involved. Then the resistance [by the fishers] becomes even greater”,* pointing to the lack of communication creating opposition between regulators and the industry. On the other hand, participant 28 who belonged to the Ecocentric group described the current older generation of fishers as *“still living in some type of “we’ll fish all we want” [paradigm]”*, exemplifying how the Ecocentric group may perceive the industry. A similar pattern is observed in other sectors of Swedish industry. For example, a study on sustainable agriculture in Sweden found two key perspectives: one with a strong environmental focus and another from farmer organisations emphasising profitable industries (Röös et al. 2023).

The split within the Japanese participants into a state-led policy prioritising perspective and a social community-focused perspective mirrors the current discussions within the seafood industry in Japan. The aforementioned Fisheries Policy reform focuses on increased efficiency and top-down control of the fisheries sector using output controls (Ganseforth 2023). It has been met with mixed reactions in Japan (e.g. Hirokawa and Thompson 2023), with many people falling in one of two camps: in support or against the new reform. Ganseforth (2023) describes the situation through a social and environmental justice lens, and explains well the interplay between the two viewpoints of bottom-up co-management in small fishery cooperatives (similar to the Community-centric group), and top-down regulation and promotion of private capital (similar to the Regulation-centric group).

With this in mind, it is notable to observe which statements split the Regulation-centric and Community-centric groups: statement 30 “Illegal fishing threatens fish stocks more than legal fishing pressure” (placed in 10 and 2, respectively), statement 38 “Consumer demand needs to be increased for eco-friendly seafood products” (9 and 4), and statement 2 “Employers need to do more to ensure health and safety of workers in the seafood industry” (7 and 3) were the statements for which the two groups had opposing opinions. It points to a focus on small-scale community with high trust as the ideal in the Community-centric group, whereas the Regulation-centric group places more importance on market-based approaches and higher-level laws and regulations. Given the intra-community trust required to make community management work (Kamiyama et al. 2018; Silva et al. 2021), it is not surprising that this features in the Community-centric paradigm. The size of the “opposing” Regulation-centric group is somewhat surprising for Japan, where informal social relations and trust are seen as important, particularly in the fishing industry (McGreevy and Akitsu 2016; Sugimoto et al. 2022). Béné et al. (2019) show how different problematisations of the food system can result in different proposed solutions. We believe that the two groups of Japanese participants reflect differences in how they problematise the seafood industry. These differences may also emerge in other contexts where national fisheries policy changes spark national debates about management strategies.

### Ecological aspects and restrictive regulations

When looking at the statements which had the most differing scores (statements 12, 39, 26 and 29 were the top most differing, see Table 2 and Figure S1 for more details), as well as reviewing the interview transcripts, it became clear that some of the most contested statements were those about ecological sustainability, in particular the statements that advocated strict restrictions on fisheries activities. Statement 12: “Bottom trawls should be banned to achieve sustainable fisheries” was the most divisive statement. Other statements that had strong reactions, seen either through the Q sort rankings or during the interviews, were statement 26 “Fishing pressure affects fish stocks more than climate change and other problems”, statement 29 “Important habitats for fish should be protected from fishing activity and fish farms”, statement 15 “Fisheries should avoid any bycatch” and statement 6 “Greenhouse gas emissions from the seafood industry should be stopped”. This is despite evidence that, for example, global seafood production can account for up to 1.5% of the global carbon dioxide emissions (Bell et al. 2018) or that protecting 21% of the ocean in highly protected marine areas would protect 87% of the range of critically endangered species (Sala et al. 2021).

Comments on these statements during the interviews fell into two patterns: against and giving nuances of the situation to support their point, and in agreement in principle but understanding that the statements were ambitious. In this sense, although the participants placed these statements at opposite ends of the Q sort, we believe that they have a clear understanding of the nuances and other positions people would take on the issue and came across quite pragmatic in the interviews. As an example, the following quotations illustrate two participants’ perspectives on the statement: “Fisheries should avoid any bycatch”. Participant 22, who ranked Statement 15 in Column 1, commented: “*Yes, we will always have bycatches, but we need to manage those bycatches in a sustainable way… And not throw them back, but make use of them*”. Participant 24, who ranked Statement 15 in Column 7, stated:

*Fisheries should avoid all bycatch. Yes, if you just kill bycatch and do nothing with it, then you need to avoid it. But if you take care of the bycatch, then it’s a different matter.*

The quotations are striking as they make the same point, even though the two participants placed the statement in very different locations in the Q sort grid. This shows the importance of the Q sort exercise to bring out differences in perspectives beyond those found during the interviews.

Participants also appealed to different dimensions of sustainability to support their point, as can be seen with participants from opposing viewpoints on bottom trawls:

*You cannot continue to destroy the habitat if you want a long-term sustainable fish stock. Also, it is a method that has extremely high bycatch of the wrong species and the wrong size. This means that not only the environment but also the stocks themselves suffer quite a lot. (Participant 27 who placed statement 12 in column 10)*

*About the bottom trawling, in the end it is about bycatch, but there are people actually making a living out of it… I do not think there is no problems with it, but if you think there are people relying on this for their living, that they are not going to survive, then what about. that? (Participant 19 who placed statement 12 in column 0)*

This points to the difficulty of implementing strict ecological regulations on the fishing industry due to the immediate effects on the social sustainability of the industry and the financial situation of the fishers. Bottom trawling is particular in that it has relative consensus that it is unique within fisheries in terms of environmental impacts, and that it causes widespread physical disturbance to the seabed (Hiddink et al. 2017; Steadman et al. 2021). At the same time, every aspect of the debate has been coloured with polarisation (Steadman et al. 2021). Even within the academic realm, there has been recent fierce debate on the effect of bottom trawling on carbon dioxide emissions, some arguing that bottom trawling releases a similar amount of greenhouse gas emissions as the agricultural industry, and some refuting this (Sala et al. 2021; Atwood et al. 2023; Hiddink et al. 2023).

This result could also point to a difference in how stakeholders conceptualised the relationship between humans and nature, and whether they see the fisheries system from an anthropocentric lens or an ecosystem-centric lens. Strict ecological regulations are likely to be supported by those that believe a healthy ecosystem is a prerequisite for society and economy whereas they are likely to be opposed by those that believe that a healthy society and economy are a prerequisite to managing environmental impacts. When working towards sustainability in the seafood supply chain, most perceive that it can only be achieved through trade-offs (Béné et al. 2019), even though some evidence suggests that economic, social and ecological sustainability are mutually reinforcing when achieved (Asche et al. 2018). Several participants reflected in agreement with Béné et al., for example in the quotation below, from a participant represented by the Ecocentric group:

*If we are to have any commercial fishing left at all, we need to have a regrowth in commercial fishing. But on the other hand, we need to make sure first and foremost that there are fish for them to actually catch. But then you end up having to find a balance of this. (Participant 27)*

### Areas of consensus

Despite the differences between the two countries, there were several topics around which perspectives aligned. All groups agreed that eating local seafood was good for sustainability—indeed, traditional local food is a current market trend in Sweden (Wallfelt 2023). It is heartening to see minimising antibiotic use on fish farms was also a consensus statement, especially when Japanese farms use significant proportions of critically and highly important antimicrobials for human medicine (Ido 2023).

There were other statements where all groups tended to have the same opinion—where all placements of the statement were on one or other side of the Q sort grid. For example, the statement about managing forests, rivers and oceans together was ranked in columns 8, 7, 6 and 9 in each group, the statement about better connecting producers and consumers was ranked in columns 10, 6, 9 and 8, and the statement advocating fewer restrictions on the fisheries sector was ranked in columns 1, 1, 3 and 1. Even though some groups answered in a more neutral way to these statements and, therefore, they were not identified as consensus statements in a statistical sense, this still indicates a potentially fruitful starting ground for negotiations towards a joint conceptualisation of sustainable seafood, as the answers given are non-confrontational. Countries that have a more similar governance and sustainability background may find more of such common ground. This could be coupled with other research, for example, Farmery et al.’s work on pathways to more sustainable seafood systems, which give actions to move towards certain aims around sustainability (Farmery et al. 2022).

Looking across categorisations of statements and how they were placed in each group, there was a slight trend in more social sustainability statements being placed in similar columns than statements to do with economic sustainability, and the similar for statements framing seafood as a common good compared to other framings. This can be represented by the Community-centric group, which could be interpreted as representing more global values since it represents participants from both case study countries. It could also be interpreted as the perspective which is less to do with country specific trends or arguments, but more to do with issues such as trust and community connections, issues that can cross country borders. Indeed, when looking at similarities across the interview data from participants in this group, the importance of trust between authorities and fishermen, and between fishermen and consumers, was a key theme. Trust, an important component of social capital (Paldam 2000), plays a role in reducing the costs of fisheries management through compliance within the fishing community and smooth sharing of information between fishers and the management authority (Grafton 2005). In his work on coastal communities, Jentoft argued that “viable fish stocks require viable fisheries communities” (Jentoft 2000), and that building communities consisted of building social capital and networks based on trust, solidarity and mutual support (Jentoft 2014). The Community-centric group appears to mirror this view, and from our results, one could conclude that the social dimension of sustainability, and the framing of seafood as a common good are the most successful aspects or arguments that can further the cause of sustainable seafood globally.

### Missing viewpoints and other caveats

In every Q method study, there is a strong possibility of missing crucial individuals who may bring a new perception (Sneegas et al. 2021). In our case, due to our condition of instruction and Q sort statements being particularly focused on sustainability, we approached individuals who were already cognisant of sustainability so that the Q sort process would be meaningful and uncover rich answers. This allowed us to gain insights into the perceptions of those knowledgeable about the subject; however, this meant that we did not interview stakeholders that do not concern themselves with sustainability. Our results, therefore, should be interpreted with this in mind and that the consensus topics represent areas of consensus for the 29 participants.

It should be noted that there were five participants whose views were not represented by any of the factors extracted. This is because the factor extraction resulted in factors that did not articulate these participants’ perspectives—their thinking did not “fit” into the four main perspectives that were found. Their views were, however, considered through using quotations from their interviews in the discussion section.

### Cross-country and cross-dimensional comparisons

There has been a recent explosion in studies using the Q method to assess differences in conceptualisations of socio-ecological phenomena and policies within environmental sustainability research (Zabala et al. 2018; Sneegas et al. 2021). However, the literature using this approach to look at sustainable seafood is limited, and our study contributes to this field. It is particularly noteworthy that we incorporated a range of dimensions of sustainability into our analysis in order to bring forth the explicit prioritisation of these dimensions and their interactions. Within the literature considering sustainable seafood, the focus is often on one or another of the dimensions, with no clear way to considering the issues simultaneously. By using Q method, we forced our participants to choose between ecological, social and economic dimensions of sustainability, and by using structured interviews, we were informed of their thinking process behind these choices. This allowed us deeper insights than if we would have used a purely quantitative or qualitative approach.

Another novelty is the cross-country nature of the study. A Q method study developed and implemented in two languages in two very different country contexts provides a unique approach to understand the conceptualisation of sustainable seafood. The study demonstrates the possibilities of using this method, and how it could be operationalised to take on more comparative research questions.

Given the globally traded nature of seafood, it is essential that we consider perspectives from countries that differ in their approaches to sustainability, and their governance strategies. We present the unique case examples of Sweden and Japan, from which we can hypothesise how in-country perspectives can be shaped, and which underlying aspects of sustainability, such as trust, may be valued across borders. In this way, we hope that others may recognise perspectives and patterns of prioritisation within the different dimensions of sustainability that exist in other countries. By presenting results from two different contexts, we provide a starting point for considering how we can move towards sustainability within the global seafood industry.

## Conclusion

Understanding how sustainable seafood is conceptualised by different actors is essential to move towards improving practices collectively. Our analysis has highlighted how embedded stakeholders’ conceptualisations of sustainability are in their cultural and market contexts, with country of origin playing the major role in how the four emerging factors were split, despite interviewing actors from a range of positions along the supply chain. We may assume that this strong context-dependency applies to other countries as well, which would be interesting to investigate in lower-income countries and in a wider range of value chain stakeholders.

The results also show how different stakeholders prioritised ecological, social and economic dimensions of sustainability, and how they frame seafood—as a commodity, human right, common good or as a living thing rather than food at all. The divisiveness of restrictive rules on the ecological impacts of seafood production showed that stakeholders perceived the relationship between human society and natural ecosystems in contrasting ways. It also highlights that stakeholders saw obvious trade-offs between social, economic and ecological dimensions of sustainability, rather than a mutual reinforcement between them.

Finally, there were areas of consensus. For example, the one group that was represented by participants from both countries prioritised social and ecological sustainability and framed seafood as a common good. This viewpoint could be interpreted as one that is more likely to be understandable from a variety of cultural and market contexts. It also embodied more relational values, prioritising statements that were concerned with building relationships between people, and between people and nature. This helps us hypothesise which values stakeholders are more likely to have in common, beyond the case study boundaries that we examined here.

Going forward, acknowledging the context-dependency of the meaning of sustainable seafood will be essential in developing approaches across country boundaries. Starting discussions from shared values, particularly those from the Community-centric perspective identified here, may facilitate more effective and transferable solutions.

## Supplementary Information

Below is the link to the electronic supplementary material.

Supplementary file1 (PDF 497 KB)

## Data Availability

The data supporting this study are available from Stockholm University with restrictions. However, the authors can provide some of the data upon reasonable request.

## Footnotes

1 Food derived from aquatic animals, plants or algae, that are captured or cultivated from the ocean, rivers, lakes, ponds, raceways and tanks.

2 https://www.wwf.se/fiskguiden/.

3 https://github.com/Abigayil/Sustainable-seafood-Q-method.

4 It is common to use the range of numbers from − 5 to + 5 in a Q sort grid. However, we received feedback from our pilot participants that they felt uncomfortable placing statements that they agreed with into the minus columns (which tends to happen if the participant agrees with more statements than they disagree with). We, therefore, decided on a purely positive number range, which does not affect the analysis and made it easier for participants to place the statements.

5 E.g. https://www.dn.se/sverige/kritik-mot-regeringens-presentation-av-havsplaner-vilseledande/, https://www.dn.se/sverige/goteborgs-stad-ville-ha-kopstopp-pa-bottentralad-fisk-fick-noja-sig-med-tjugo-procent/.
