# Supplementary material for: What does “sustainable seafood” mean to seafood system actors in Japan and Sweden?
Source: Ambio. 2025 Jan 2;54(6):1010–25. doi: 10.1007/s13280-024-02122-4 (PMC12055706; doi:10.1007/s13280-024-02122-4)
Supplement: Supplementary file 1 — Supplementary file1 (PDF 497 KB) [file 13280_2024_2122_MOESM1_ESM.pdf]

Ambio

Supplementary Information

This supplementary information has not been peer reviewed.

Title: **What does “sustainable seafood” mean to seafood system actors in Japan and Sweden?**

Table S1 List of organisations associated with fisheries, aquaculture and seafood in Sweden and Japan, used to download resources to create the Q-set of statements

Actor/organisation	English Translation	Explanation
SFPO - Sveriges Fiskares Producentorganisation	Swedish Fisheries Producer Organisation	
SPF-PO - Swedish Pelagic Federation Producentorganisation	Swedish Pelagic Federation Producer Organisation	
Insjöfiskarena AB<br>Svenska Insjöfiskarenas Centralförbund	Swedish Inland Fishermen's Federation	
Matfiskodlarna	Food fish farmers	Aquaculture producer organisation in Sweden
Skillinge Fisk-Impex AB		One example of a seafood processor in Sweden
Orkla		A large Swedish seafood processor, brand owner
ICA		Largest Swedish supermarket
Coop		Second largest Swedish supermarket
Axfood		Third largest Swedish grocers
WWF Sverige	WWF Sweden	
Naturskyddsföreningen	Swedish Nature Conservation Society	
KRAV		Swedish environmental certification
Havs och Vattenmyndigheten	Swedish Agency for Marine and Water Management	
Jordbruksverket	Swedish Board of Agriculture	

Actor/organisation	English Translation	Explanation
Fiskbranschens Riksförbund	National Association of the Fish Industry	
Svensk Fisknäring and Sjömatsfrämjandet	Swedish fisheries and Seafood promotion	Swedish fisheries magazine and Seafood promotion organisation
Svenssons Nyheter Njord	Njord news	Sweden’s largest newspaper about commercial fishing
Livsmedelsverket	Swedish food agency	
Axfoundation		Independent, non-profit organisation with hands on projects on sustainable food
全漁連 Zengyoren	National Federation of Fisheries Co-operative Associations	
東京都水産物卸売業者協会 Tokyoto Suisanbustu Oroshiurigyousya kyoukai	Tokyo city seafood wholesaler association	
中央魚類 Chuo gyorui (Marunaka)		One of the wholesalers at Toyosu
東都水産 Tohto suisan		One of the wholesalers at Toyosu
築地魚市場 Tsukiji Uoichiba		One of the wholesalers at Toyosu
第一水産 Dai-ichi Suisan		One of the wholesalers at Toyosu
AEON		Largest Japanese retailer
Ito Yokado		Large Japanese retailer
COOP（生協）		Large Japanese retailer

Actor/organisation	English Translation	Explanation
Seafood Legacy		Japanese NGO
MEL Marine Eco-Label Japan		Domestic marine eco-label
Blue Seafood Guide Japan		
水産庁 Suisancho	Japanese Fisheries Agency	
Minato Newspaper		
Nissui		Second largest Japanese seafood company
Maruha -Nichiro		Largest Japanese seafood company
Kyokuyo		Third largest Japanese seafood company
Hokkaido Gyoren	Hokkaido Fisheries Co-operative Association	One of the largest regional fisheries co-operative associations
全国スーパーマーケット協会 Zenkoku Supermarket Kyokai	Country-wide Supermarket Association	

Table S2 Q-set of 40 Q sort statements translated into Swedish (left) and Japanese (right), together with the condition of instruction for both languages at the top of the table

ID	Vad är viktigt för att uppnå hållbar sjömat?	持続性が担保された水産物の提供に至るための重要な課題は何でしょうか？
1	Odlad sjömat bör inte utfordras med sådant som människor kan äta	養殖される魚には、人間が食べられるような食材を与えるべきではない
2	Arbetsgivare inom sjömatsindustrin behöver göra mer för att säkerställa de anställdas hälsa och säkerhet	水産業に従事する労働者の安全と健康を確保するために、雇用主はもっと努力するべきだ
3	Sjukdoms- och parasitutbrott är de viktigaste problemen för vattenbruket	養殖においては、魚病や病害虫の問題が最重要課題だ
4	Spårbarhet är avgörande för hållbar sjömat	トレーサビリティーは水産物の持続可能性に欠かせない
5	Småskaliga fiskare bör få mer statligt stöd än det industriella fisket	小規模な漁業者は大規模な漁業者よりも国から多く支援を受けるべきだ
6	Växthusgasutsläppen från sjömatsindustrin bör stoppas	水産業からの温室効果ガスの排出は止めるべきだ
7	Antibiotikaanvändning bör minimeras i fiskodlingar	養殖場での抗生物質の使用は最小限にとどめるべきだ
8	Fiskodlingar bör vara landbaserade för att öka hållbarheten	環境への負荷を減らすために、完全陸上養殖をすべきだ
9	Fisketrycket kan tillåtas öka även om uppgifter om fiskbeståndets saknas	資源状態のデータが不足している中で、漁獲圧を高めても問題はない
10	Ökad uppföljning, kontroll och övervakning behövs för att säkerställa att fiskare följer regleringarna	漁業者が規制を遵守するよう、監視、制御やモニタリングを強化しなければならない
11	Det är mer hållbart att äta lokal sjömat	地元の水産物を食べたほうが環境にやさしい
12	Bottentrålning bör förbjudas för att uppnå hållbart fiske	持続可能な漁業を達成するために底引き網の利用を禁止するべきだ
13	Begränsningar av fiskeansträngning är mer effektivt för att reglera fisketrycket än fiskekvoter	漁獲圧の管理のためには、漁獲努力量の制限（入口管理）のほうが数量管理（出口管理）より効果的だ
14	Vi bör äta fler fiskarter för att minska trycket på de mest populära arterna	最も需要が高い魚種の漁獲圧を減少させるため、もっと多くの魚種を食べるべきだ
15	Fisket bör undvika all bifångst	漁業による混獲はどの種であり避けるべきだ
16	Den kulturella traditionen kring ätande av sjömat är avgörande för hållbarhet	魚食文化などの伝統は持続可能性に欠かせない
17	Låg lönsamhet är fiskets största problem	収益性の低さが漁業の主な問題だ
18	Fiskeförvaltningen bör antas i samarbete med fiskare	漁業管理の意思決定は漁業者と一緒に行うべきだ
19	Kvinnor och minoriteter bör ges fler möjligheter att delta i sjömatsindustrin	女性やマイノリティが水産業に参加する機会をもっと増やすべきだ

20	Det bör göras lättare för yngre generationer att bli yrkesfiskare	若い世代が漁業者になりやすいようにすべきだ
21	Miljöcertifieringar är den bästa indikatorn för hållbar sjömat	水産物の認証制度は水産物の持続可能性を示すためのベストな方法だ
22	Fiskeförvaltningen bör vara mindre restriktiv för fiskerisektorn	漁業管理はもっと緩和されるべきだ
23	Att skapa mervärde genom en förbättrad fisk-processindustri är nyckeln till hållbarhet	加工産業を改善して高付加価値化することが持続可能性のために必要だ
24	Vi bör minska vår inhemska fiskkonsumtion av hållbarhetsskäl	持続可能性のために、国内の魚の消費量を減らすべきだ
25	Det är bättre att odla sjömatsarter som inte behöver utfodras, t.ex ostron	カキのような無給餌養殖は給餌養殖より望ましい
26	Fisketrycket påverkar fiskbestånden mer än klimatförändringar och andra problem	漁獲圧は気候変動などの問題よりも資源量に悪影響を与える
27	Individuella överlåtbara kvoter är ett bra sätt att uppnå ett hållbart fiske	ITQ（個別譲渡可能割当制）は漁業を持続可能にする良い方法だ
28	Det är viktigt att minska antalet rymningar från fiskodlingar för att säkerställa friska vilda populationer	養殖場からの魚の流出を減少させ、健全な野生個体群を確保することは重要だ
29	Viktiga livsmiljöer för fisk bör skyddas från fiske och fiskodlingar genom marina skyddade områden	魚にとって重要な生息地は、漁業活動や養殖場から保護区として守るべきだ
30	Olagligt fiske hotar fiskbestånden mer än det lagliga fisketrycket	違法漁業は合法的な漁業よりも水産資源に悪影響を及ぼしている
31	Fiskare bör skyddas från kostnadsökningen för att bedriva fiske	漁業者は、漁業経営における価格高騰から保護されるべきだ
32	Att öka effektiviteten genom högpresterande fiskefartyg och fiskeutrustning kommer att öka fiskets hållbarhet	高性能の漁船や漁業用機器による効率化で、漁業の持続可能性が高まる
33	Hotade arter kan fiskas så länge det finns en återhämtningsplan för beståndet och regler för fiskeförvaltning	絶滅危惧種は、資源回復計画や漁業管理ルールがある限り、漁獲できる
34	Relationen mellan fiskesamhällen och havet bör skyddas	漁村と海とのつながりは守るべきである
35	Skogar, floder och hav bör förvaltas tillsammans för att ta hänsyn till återkopplingar dem emellan	森、川、海はそのつながりを考慮し、一緒に管理するべきだ
36	Kopplingen mellan producenter och konsumenter av sjömat bör förbättras för att fördjupa ömsesidig förståelse	水産物の生産者と消費者は相互理解を深め、より良い関係を築くべきだ
37	Det är nödvändigt att stärka fiskesamhällena som helhet, inte bara de ekologiska aspekterna av fisket	漁業の生態学的側面だけでなく、漁村全体の活性化が必要である
38	Konsumenternas efterfrågan för miljövänlig sjömat måste öka	持続性を担保された水産物に対する消費者需要の拡大が必要である
39	Regelverk för vattenbruk måste förenklas för att göra det enklare att starta odlingar	養殖場の設置をより簡単にするために、規制は簡素化されるべきだ
40	Om fisket är lagligt är det hållbart	漁業が合法であれば、持続可能でもある

Table S3 Reasoning behind each statement's categorisation into one of the three dimensions of sustainability and the four framings of seafood.

ID	Statements	Ecological /Social /Economic	Framing	Reasoning
1	Farmed seafood should not be fed from ingredients that humans could eat	Ecological	Human right	There are two angles to the statement, one which is about limiting ecological impacts of farming fish, and one which is about fair access to fisheries resources. We therefore place the statement in both Ecological sustainability and a Human rights framing.
2	Employers need to do more to ensure health and safety of workers in the seafood industry.	Social	Human right	The statement is about limiting detrimental social impacts of the seafood industry (social sustainability), and an absence of human exploitation (human rights).
3	Disease and parasite outbreaks are the most important issues in aquaculture	Ecological	Biosphere-centric	The statement is only about limiting ecological impacts of the seafood industry.
4	Traceability is essential for sustainable seafood	Other	Human right	Traceability can be useful for any of the three sustainability dimensions (therefore placed in none of them), and is about transparency of information about food resources (human rights).
5	Small scale fishers should receive more support from the state than industrial fishing	Social	Common good	The statement is about the social sustainability of small scale fishers and is about food embedded in regional contexts (common good).
6	Green house gas emissions from the seafood industry should be stopped	Ecological	Biosphere-centric	The statement is only about limiting ecological impacts of the seafood industry.
7	Antibiotic use should be minimised in fish farms	Ecological	Biosphere-centric	Excessive antibiotic use can have effects on the environment and human health. Here we categorise the statement based on the effects is can have within the seafood system (primarily environmental), but this statement could also be classified as "Social" and "Human right".
8	Fish farms should be land-based to increase sustainability	Ecological	Biosphere-centric	The statement is only about limiting ecological impacts of the seafood industry.
9	Fishing pressure can be increased even in the absence of data on fish stocks	Ecological	Biosphere-centric	The statement is only about limiting ecological impacts of the seafood industry.

10	Increased monitoring, control and surveillance is needed to ensure fishers comply with regulations	Social	Human right	The statement is about the social aspects of the seafood industry, and is about fair compliance with regulations as well as control of rights depending on who is implementing the surveillance (human rights).
11	It is better for sustainability to eat local seafood	Social	Common good	The statement is about supporting local small scale fisheries by eating local seafood. In this sense it focuses on the social sustainability of the seafood and food embedded in regional contexts (common good).
12	Bottom trawls should be banned to achieve sustainable fisheries	Ecological	Biosphere-centric	The statement is only about limiting ecological impacts of the seafood industry.
13	To manage fishing pressure, limits on fishing effort are more effective than fishing quotas	Social	Human right	The context from which this statement comes from is the discussion about limiting fishing effort (input control) versus limiting fish landings (output control). Fishing effort is more easily controlled by the fishers themselves whereas quotas on fish landings are often imposed by a higher authority, or the state. Therefore this statement is about the decision making about fisheries management (social sustainability), and democratic participation in this management (human rights).
14	We should eat more fish species to take the pressure off the most popular species	Ecological	Commodity	The statement is about reducing ecological impacts, but does this through a commodity-lens on seafood i.e. consumption pressure being the main driving force that can drive sustainability.
15	Fisheries should avoid any bycatch	Ecological	Biosphere-centric	The statement is only about limiting ecological impacts of the seafood industry.
16	Cultural traditions around eating fish is essential for sustainability	Social	Common good	The statement is about how cultural traditions can or cannot merge with sustainability (social sustainability) and about how food is embedded in regional contexts (common good).
17	Low profitability is the major problem of fisheries	Economic	Commodity	The statement is about economic sustainability of the seafood industry, and focuses on profitability (commodity).
18	Fisheries management should be decided together with fishers	Social	Common good	This statement is about the decision making around fisheries management (social sustainability), and focuses on decentralised or polycentric governance (common good).

19	Women and minorities should be given more opportunities to take part in the seafood industry	Social	Human right	The statement is about increasing the inclusivity of the seafood industry (social sustainability) and allowing equitable access to resource production (human rights).
20	It should be made easier for younger generations to become fishers	Social	Human right	The statement is about increasing the inclusivity of the seafood industry (social sustainability) and allowing equitable access to resource production (human rights).
21	Eco-certifications are the best indicator for sustainable seafood	Ecological	Commodity	The statement is about reducing ecological impacts (ecological sustainability) through a commodity-lens on seafood i.e. market-based approaches are the main driving force that can drive sustainability.
22	Fisheries management should be less restrictive for the fisheries sector	Social	Human right	This statement is about fisheries management (social sustainability), and includes ideas about what should be considered fair (or restrictive) access to resources (human rights).
23	Adding value through an improved processing industry is the key to sustainability	Economic	Commodity	The statement is about increasing the profitability of the seafood industry (economic sustainability) and highlights the economic value of the seafood as its main characteristic (commodity).
24	We should reduce our domestic fish consumption for sustainability	Social	Commodity	The statement is about a social strategy for sustainability (social sustainability) using a commodity-lens on seafood i.e. changes in consumption pressure is the main tool towards sustainability.
25	It is better to farm aquatic species that do not need feeding, such as oysters	Ecological	Biosphere-centric	The statement is only about limiting ecological impacts of the seafood industry.
26	Fishing pressure affects fish stocks more than climate change and other problems	Ecological	Biosphere-centric	The statement is only about the ecological impacts of the seafood industry.
27	Individual Transferable Quotas are a good way to achieve sustainable fisheries	Economic	Commodity	This statement focuses on Individual Transferable Quotas which have been described in the literature as an approach to optimise fisheries output so that profitability is increased and effort is decreased. We therefore place it under economic sustainability and commodification of seafood.
28	Reducing the escape of fish from fish farms to ensure healthy wild populations is important	Ecological	Biosphere-centric	The statement is only about limiting ecological impacts of the seafood industry.

29	Important habitats for fish should be protected from fishing activity and fish farms	Ecological	Biosphere-centric	The statement is only about limiting ecological impacts of the seafood industry.
30	Illegal fishing threatens fish stocks more than legal fishing pressure	Social	Human right	This statement is about a social problem in seafood which is about undemocratic access to the resources (human rights).
31	Fishers should be protected from the rising prices of operating a fishery	Economic	Commodity	The statement is about economic sustainability of the seafood industry, and focuses on profitability (commodity).
32	Enhancing efficiency through high-performance fishing vessels and fishing equipment will increase the sustainability of the fishery	Economic	Human right	The statement is about economic sustainability of the seafood industry, but also about increasing access to the fisheries resources (human rights).
33	Endangered species can be fished as long as there is a stock recovery plan and fisheries management rules	Ecological	Biosphere-centric	The statement is only about limiting ecological impacts of the seafood industry.
34	The connection between fisheries communities and the ocean should be protected	Social	Common good	The statement is about a social aspect of sustainability and about how food is embedded in regional contexts (common good).
35	Forests, rivers and the ocean should be managed together to account for the feedbacks between them	Ecological	Biosphere-centric	The statement is only about limiting ecological impacts of the seafood industry.
36	Seafood producers and consumers should be better connected with deepened mutual understanding	Social	Common good	The statement is about a social aspect of sustainability, and cooperation and mutual understanding which are important aspects of ideas around common good management.
37	It is necessary to revitalise fishing communities as a whole, not just the ecological aspects of fisheries	Social	Common good	The statement is about the social aspect of the seafood industry and focuses on fisheries communities and how food is embedded in regional contexts (common good).
38	Consumer demand needs to be increased for ecofriendly seafood products	Economic	Commodity	The statement is about the profitability and economic sustainability of eco-friendly seafood products and uses a commodity-lens on seafood i.e. consumption pressure is the main driving force that can drive sustainability.
39	Regulation of aquaculture must be simplified to make it easier to set up farms	Economic	Human right	The reasoning behind this statement is primarily to do with the profitability and economic incentives in the seafood industry (economic sustainability) but also has to do with the right to access food production (human rights).

40	If the fishery is legal, it is also sustainable	Other	Human right	This statement does not fall under any of the three dimensions of sustainability, but aligns with the idea that the state can act as the main guarantor of the right to food (human rights).

Interview guide:

1. Where is the line between “agree” and “disagree” for you?
2. Can you explain why you placed these particular statements under “most agree”/ “most disagree”?
3. What do you think are the solutions to the statements which you most agree with?
4. Statements which you thought a lot about, or those that were placed in slightly odd places:

a. What did you consider when you placed them?

5. Where there any statements that were not clear?
6. Where there any statements that could be interpreted in different ways?

a. How did you interpret them?

7. Where there any statements that were missing in your opinion?

a. If you were to write your own statement, what would you write about?

b. Where would you place it in the grid?

Table S4 Eigenvalues and percentage of study variance explained by each factor in a 4 factor extraction

	Eigenvalues	Percentage of study variance explained
Factor 1	6.03	20.78
Factor 2	4.28	14.75
Factor 3	3.65	12.57
Factor 4	3.20	11.03

Table S5 Correlations between each factor

	Factor 1	Factor 2	Factor 3	Factor 4
Factor 1	1	0.34	0.38	0.36
Factor 2	0.34	1	0.14	0.14
Factor 3	0.38	0.14	1	0.42
Factor 4	0.36	0.14	0.42	1

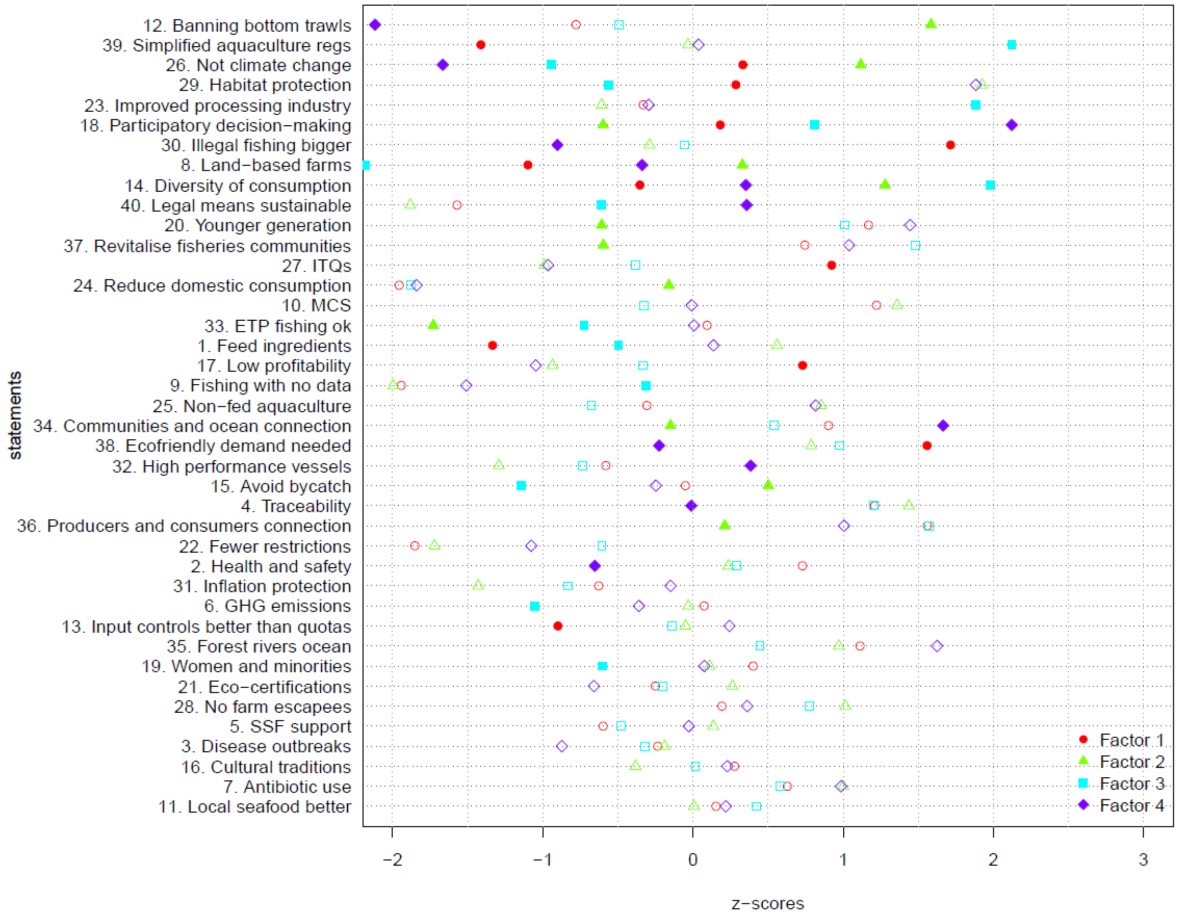

Figure S1 Plot of z-scores for each statements for each factor. Statements are ranked from most distinguishing at the top, to most consensus at the bottom. Statements are shortenings of the original statements, with corresponding statement ID number. Solid symbols show which statements are distinguishing statements for each factor. The z-score is the weighted average score given to a statement by participants that are loaded on each factor

Sensitivity analysis to check the variation in opinion within participants from the same country

Summary:

Three factors were extracted from each country. The three factors extracted from the Japanese subset explained 67% of the data variance in that subset. All Japanese participants were represented by the three factors, with 6 Q sorts loaded on the first factor, and 4 Q sorts loaded on both the second and third factors. The three factors extracted from the Swedish subset explained 61% of the data variance in that subset, and all but one Swedish participant were represented by the factors. 5 Q sorts are loaded onto both the first and third factors, and 4 Q sorts are loaded onto the second factor.

Table S6 Table showing number of Q sorts loaded, eigenvalues and percentage of explained variance for the resulting factors from the sensitivity analysis conducted on Q sorts from each country.

Japan country subset	Factor 1	Factor 2	Factor 3
Number of Q sorts loaded on each factor			
Eigenvalues	3.61	2.98	2.80
Percentage of explained variance	25.76	21.26	20.00
Sweden country subset			
Number of Q sorts loaded on each factor			
Eigenvalues	3.79	2.96	2.33
Percentage of explained variance	25.24	19.73	15.55

Mapping of country-specific factors onto factors extracted from overall sample:

Table S7 Table showing which factors each participant were related to (i.e. flagged) within the main analysis, and the country-specific sensitivity analysis.

	**Participants from Japan**	
Participant ID	Main analysis factor number	Country-specific factor number for the same participant
1	1	1
13	1	1

14	1	1
18	1	1
26	1	1
16	1	2
19	1	2
7	1	3
11	1	3
2	4	3
3	4	3
20	none attributed	1
4	none attributed	2
10	none attributed	2
	**Participants from Sweden**	
	Main analysis factor loadings	Country-specific factor loadings for the same participant
8	2	1
15	2	1
17	2	1
23	2	1
27	2	1
6	3	2
9	3	2
22	3	2
25	3	2
28	3	3
21	4	3
29	4	3
12	4	none attributed
5	none attributed	3
24	none attributed	3

In brief, Factor 1 in the main analysis was split into three factors in the sensitivity analysis for Japan, with those who came under Factor 4 in the main analysis feeding into the third factor in the sensitivity analysis. From this we predict that the third factor for the sensitivity analysis for Japan is similar to Factor 4 in the main analysis.

For Sweden, Factor 2 exclusively maps onto the first factor in the sensitivity analysis, so we can hypothesise that the content of the two factors is similar. Factor 3 almost completely maps onto the second factor in the sensitivity analysis, which implies similarity between these two factors as well. The third factor of the sensitivity analysis includes two participants who came under Factor 4 in the

main analysis, one participant from Factor 3, and two participants who did not have an attributed factor in the main analysis. We can hypothesise that this factor is similar to Factor 4 but also a mixture of viewpoints from other participants.
